# Interval Colorectal Cancers in a Fecal Immunochemical Test–Based Screening Program

**DOI:** 10.1001/jamanetworkopen.2025.23441

**Published:** 2025-07-28

**Authors:** Wen-Feng Hsu, Uri Ladabaum, Chiu-Wen Su, Chen-Yang Hsu, Amy Ming-Fang Yen, Sam Li-Sheng Chen, Tsui-Hsia Hsu, Li-Ju Lin, Yi-Chia Lee, Ming-Shiang Wu, Hsiu-Hsi Chen, Han-Mo Chiu

**Affiliations:** 1 Department of Internal Medicine, National Taiwan University Hospital, Taipei, Taiwan; 2 Division of Gastroenterology and Hepatology, Stanford University School of Medicine, Stanford, California; 3 Graduate Institute of Epidemiology and Preventive Medicine, College of Public Health, National Taiwan University, Taipei, Taiwan; 4 School of Oral Hygiene, College of Oral Medicine, Taipei Medical University, Taipei, Taiwan; 5 Health Promotion Administration, Ministry of Health and Welfare, Taipei, Taiwan

## Abstract

**Importance:**

Interval colorectal cancers (CRCs) can occur after a negative result on a primary screening test or after a colonoscopy for a positive screening test result without the diagnosis of CRC.

**Objective:**

To compare the burden, incidence, mortality, and long-term survival of interval CRC after a fecal immunochemical test (post-FIT interval CRC) and colonoscopy (postcolonoscopy interval CRC) in the FIT-based Taiwan CRC Screening Program.

**Design, Setting, and Participants:**

This cohort study analyzed participants (aged 50-74 years) in the Taiwan CRC Screening Program who completed at least 1 FIT between January 1, 2004, and December 31, 2012. Two cohorts were created: one including individuals with a negative FIT result, and the other comprising individuals with a positive FIT result followed by a negative colonoscopy result. Post-FIT interval CRC or postcolonoscopy interval CRC and their survival status were identified and verified via linkage to Taiwan Cancer Registry and Taiwan Death Registry through December 31, 2019. Data analysis was performed between January 2004 and December 2019.

**Exposures:**

Participants were screened using FIT, with follow-up colonoscopy offered to individuals who tested positive. The main exposures of interest were (1) negative FIT vs positive FIT result with a negative colonoscopy result, and (2) negative FIT and positive FIT results with a negative colonoscopy result further stratified by hospital-level adenoma detection rate (ADR). Low was defined as less than 40% ADR, middle was between 40% and less than 65% ADR, and high was 65% or higher ADR.

**Main Outcomes and Measures:**

The primary outcomes were the incidence, CRC-specific mortality, and survival rates for post-FIT interval CRC and postcolonoscopy interval CRC.

**Results:**

A total of 4018 patients with interval CRC (mean [SD] age at diagnosis, 62.8 [5.9] years; 2118 males [52.7%]) were included in the analysis. Among 15 386 total CRCs in the program, 10 515 (68.3%) were screen-detected CRCs and 4018 (26.2%) were interval CRCs. Of these interval CRC cases, 2782 (18.1%) were post-FIT interval CRCs and 1236 (8.1%) were postcolonoscopy interval CRCs. Compared with post-FIT interval CRC detected after a negative FIT result, postcolonoscopy interval CRC incidence (0.75 [95% CI, 0.71-0.79] per 1000 person-years vs 0.09 [95% CI, 0.09-0.10] per 1000 person-years) and mortality (0.12 [95% CI, 0.11-0.14] per 1000 person-years vs 0.02 [95% CI, 0.02-0.03] per 1000 person-years) after follow-up colonoscopy were higher, with adjusted hazard ratios (AHRs) of 7.06 (95% CI, 6.35-7.57) and 5.04 (95% CI, 4.33-5.85), respectively. Hospital-level ADR was inversely associated with postcolonoscopy interval CRC incidence (high- vs low-ADR group AHR, 0.26; 95% CI, 0.20-0.36) and mortality (AHR, 0.28; 95% CI, 0.19-0.41). However, among patients with postcolonoscopy interval CRC, CRC-specific death was higher in the high-ADR vs low-ADR setting (AHR, 1.89; 95% CI, 1.04-3.43).

**Conclusions and Relevance:**

This cohort study found that interval CRC incidence and mortality were higher after follow-up colonoscopy than after negative FIT result, which suggests that individuals with a positive FIT result undergoing a colonoscopy exhibit a much higher risk for CRC. Survival after the diagnosis of CRC in the high-ADR subgroup was worse, suggesting a ceiling effect for current colonoscopic techniques and highlighting a possible interplay between procedural and biological factors.

## Key Points

**Question:**

In a fecal immunochemical test (FIT)–based colorectal cancer (CRC) screening program, what are the incidence, CRC-specific mortality, and survival of interval CRC?

**Findings:**

In this cohort study of 4018 persons with interval CRCs, the high adenoma detection rate (ADR) was associated with lower postcolonoscopy interval CRC incidence and mortality compared with the low-ADR and middle-ADR groups; however, the survival for postcolonoscopy interval CRC was worse in the high-ADR group.

**Meaning:**

The worse survival of postcolonoscopy interval CRC in the high-ADR group suggests a ceiling effect for current colonoscopic techniques and highlights the possible interplay between procedural and biological factors.

## Teaser

This cohort study examines the incidence, mortality, and survival associated with interval CRC detected from a fecal immunochemical test vs a follow-up colonoscopy and stratified by hospital-level adenoma detection rate.

## Introduction

The fecal immunochemical test (FIT) is the most widely used method for organized colorectal cancer (CRC) screening.^1^ While FIT screening effectively reduces CRC mortality, interval CRCs may occur after a negative FIT result (post-FIT interval CRC) or after colonoscopy following a positive FIT result (postcolonoscopy interval CRC), affecting the screening program’s effectiveness.^2,3,4,5^

In the FIT-based Taiwan CRC Screening Program, postcolonoscopy interval CRC incidence is higher than post-FIT interval CRC due to the greater CRC risk in individuals with a positive FIT result.^6,7^ Suboptimal colonoscopy quality is the primary factor contributing to postcolonoscopy interval CRC, with overlooked neoplasms accounting for 60% to 80% of cases,^8^ and the colonoscopist’s adenoma detection rate (ADR) is inversely associated with future risk of CRC, advanced-stage CRC, and related mortality.^9,10^ Furthermore, improving ADR reduces the risk of postcolonoscopy interval CRC and related death.^11,12^ In FIT-based programs, ADR is also inversely associated with postcolonoscopy interval CRC risk.^13,14^

Both post-FIT interval CRC and postcolonoscopy interval CRC are often diagnosed at advanced stages, but their long-term prognosis remains unclear due to limited cases in individual studies. Consequently, findings from larger cohorts in FIT-based screening settings are eagerly awaited.^6,7,15^ When considering the long-term outcome of postcolonoscopy interval CRC after a colonoscopy performed in a setting with diverse ADR levels, it is plausible to conjecture that postcolonoscopy interval CRC in a high–ADR setting may represent rapidly progressing neoplasms. However, this hypothesis remains unexplored. This study aimed to compare the burden, incidence, mortality, and long-term survival of post-FIT interval CRC and postcolonoscopy interval CRC in the FIT-based Taiwan CRC Screening Program.

## Methods

The National Taiwan University Hospital Research Ethics Committee approved this cohort study and waived the informed consent requirement because this was a retrospective analysis of a large, nationwide database. We followed the Strengthening the Reporting of Observational Studies in Epidemiology (STROBE) reporting guideline.^16^

### Taiwan CRC Screening Program and the Study Population

The Taiwan CRC Screening Program, launched in 2004, offers biennial FIT for individuals aged 50 to 69 years initially and was extended to those aged 74 years in 2013. Eligible persons provided 1 stool sample with a 20-μg hemoglobin/g cutoff for FIT positivity.^6^ Screening program data are linked to the Taiwan Cancer Registry and Death Registry, both covering 99% of the population,^17^ for identifying interval CRC and assessing survival status.^2,18^

Individuals with positive FIT results are referred to the nearest accredited hospital for colonoscopy if they obtained screening kits from health bureaus or clinics, while those who received kits from hospitals typically underwent colonoscopy at the same facility. According to Taiwan’s CRC screening guidelines, individuals with positive FIT result are advised to undergo colonoscopy within 6 months, as delays beyond this period are associated with increased CRC risk and more advanced disease.^19,20^ For this study, individuals who did not undergo follow-up colonoscopy within 6 months were excluded to ensure consistency with clinical guidelines and improve the reproducibility of cohort definitions. We defined 2 cohorts based on the FIT-based screening program. The first cohort included individuals with a negative FIT result. The second cohort comprised individuals with a positive FIT result followed by a negative colonoscopy result. Interval CRC and related deaths were identified and verified through December 31, 2019.

As individual endoscopist-level ADR data were unavailable during the study period, hospital-level ADRs were used instead. ADR calculation details are provided in eMethods in Supplement 1. Briefly, ADR was categorized into 3 groups: low ADR, middle ADR, and high ADR. The low-ADR group was defined as colonoscopy settings with an ADR less than 40%, consistent with the benchmark of Taiwan’s CRC screening guidelines.^19^ The middle-ADR group encompassed colonoscopy settings with an ADR ranging from 40% to less than 65%. The high-ADR group was defined as colonoscopy settings with an ADR of 65% or higher, following the criteria for high detectors in positive FIT colonoscopy results outlined in previous studies from Canada and the Asia-Pacific.^21,22^ If a hospital’s ADR level changed over time, it was recategorized into the new ADR group, and the person-years of individuals with a positive FIT result who underwent a colonoscopy at this hospital during this period contributed to the person-years of the corresponding ADR group.

### Definition of Interval CRC Categories

The definitions of interval CRC were based on the World Endoscopy Organization’s international expert panel recommendations.^4,5^ For individuals with negative FIT result, follow-up began on the date of FIT administration. Post-FIT interval CRC was defined as CRC diagnosed after a negative FIT result but before the next scheduled FIT screening, with diagnosis dates confirmed through the linked Cancer Registry. For individuals with positive FIT and negative colonoscopy result, follow-up began on the date of colonoscopy. Postcolonoscopy interval CRC was defined as CRC diagnosed within the recommended surveillance interval after an index colonoscopy. Specifically, CRCs diagnosed within 3 years after detecting advanced adenomas, high-risk sessile serrated lesions (SSLs), or 3 or more nonadvanced adenomas; within 5 years after detecting 1 to 2 nonadvanced adenomas; or within 10 years after a colonoscopy without detecting a neoplastic lesion were classified as postcolonoscopy interval CRC. Cases diagnosed beyond these respective intervals were not considered interval cancers.^23,24^ An advanced adenoma was defined as an adenomatous lesion meeting at least 1 of the following: size 10 mm or larger, villous component, or high-grade dysplasia.^25^ A high-risk SSL was defined as 1 lesion 10 mm or larger or with dysplasia.^23^

### Stage and Anatomical Site of Interval CRC

Interval CRC cases were categorized into 4 stages (stages I-IV), following the sixth edition (2004-2009), seventh edition (2010-2017), and eighth edition (2018-2019) of the American Joint Committee on Cancer and the Union for International Cancer Control tumor-node-metastasis staging system.^26^ For the anatomical site of interval CRC, the proximal colon includes segments up to the splenic flexure, while the distal colon includes the rectum and segments beyond the splenic flexure.

### Statistical Analysis

#### Incidence, CRC-Specific Mortality, and Attributable Proportion of Interval CRC

The attributable proportions of each category of interval CRC among all CRC cases detected within the screening program were calculated by dividing the number of post-FIT interval CRC or postcolonoscopy interval CRC by the total number of CRC cases detected in the screening program. Proportions were compared using the χ^2^ test for statistical significance, with *P* < .05 considered statistically significant. Rates were calculated and expressed per 1000 person-years, with person-years determined by summing each individual’s follow-up time. For interval CRC incidence, the follow-up duration started from the date of FIT (for post-FIT interval CRC) or index colonoscopy (for postcolonoscopy interval CRC) to interval CRC diagnosis or December 31, 2019, whichever occurred first. For CRC-specific mortality, the follow-up duration was calculated similarly to the incidence analysis, beginning from the date of FIT (for post-FIT interval CRC) or index colonoscopy (for postcolonoscopy interval CRC) to CRC-specific death or December 31, 2019, whichever occurred first.

CRC-specific death data were identified from the Taiwan Death Registry linked to the Taiwan Cancer Registry using *International Classification of Diseases, Ninth Revision* codes 153 to 154 and *International Statistical Classification of Diseases and Related Health Problems, Tenth Revision* codes C18 to C20, distinguishing CRC from other causes. The cumulative incidence and CRC-specific mortality for postcolonoscopy interval CRC across different ADR groups were estimated using the cumulative incidence function to account for competing risks. Differences across strata were compared using the Gray test. Cause-specific hazard models were constructed to compare the incidence and CRC-specific mortality of post-FIT interval CRC and postcolonoscopy interval CRC. Analyses adjusted for age and sex estimated the adjusted hazard ratios (AHRs) with 95% CIs, with the same regression model used to compare CRC incidence and mortality across different ADR groups for postcolonoscopy interval CRC. Furthermore, a sensitivity analysis was conducted using an alternative approach, subdistribution hazard models, to account for competing risks.

#### Survival Outcomes in Post-FIT and Postcolonoscopy Interval CRC

Survival status was explored and analyzed for different categories of interval CRC. To ensure sufficient observation time, we identified individuals with interval CRC up to December 31, 2012, and followed up on their survival status through December 31, 2019. Survival time was defined as the time from the date of diagnosis to the date of CRC-specific death or December 31, 2019, whichever came first. The Kaplan-Meier curve and log-rank test were used to compare survival status.

Cox proportional hazards regression model compared CRC-specific death risk between post-FIT interval CRC and postcolonoscopy interval CRC across ADR groups, adjusting for age, sex, the interval from FIT or colonoscopy to interval CRC diagnosis, location, and stage. Furthermore, we conducted sensitivity analyses accounting for the follow-up durations and stratified analysis by index colonoscopy findings. All statistical analyses were performed between January 2004 and December 2019 with SAS 9.4 (SAS Institute).

## Results

### Demographics, Incidence, and CRC-Specific Mortality 

**Figure 1.**
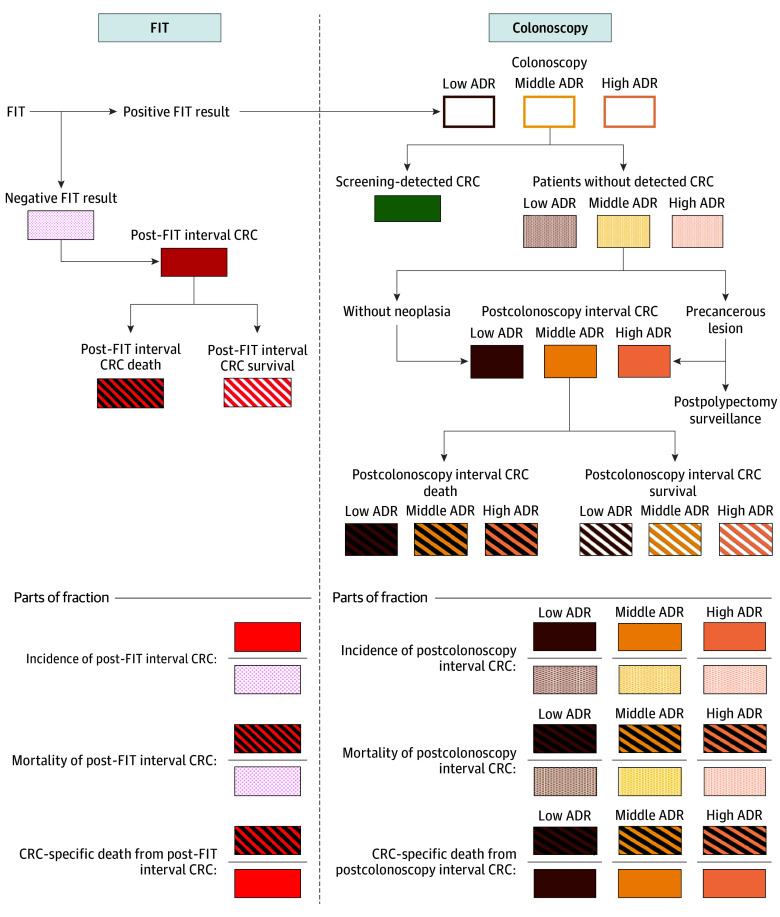
Diagrammatic Sketch of the Fecal Immunochemical Test (FIT)–Based Taiwan Colorectal Cancer (CRC) Screening Program and Its Relevant Outcome Measures Low adenoma detection rate (ADR) is defined as less than 40%, middle ADR as 40% to less than 65%, and high ADR as 65% or higher.

From January 1, 2004, to December 31, 2012, 2 982 888 individuals in Taiwan participated in FIT screening at least once, and 172 536 individuals underwent a colonoscopy following a positive FIT result. The mean (SD) time from a positive FIT result to a colonoscopy was 47.6 (26.3) days. Figure 1 shows the diagrammatic sketch of the screening program and the relevant outcomes.

Among the 15 386 CRCs diagnosed in the program, 10 515 (68.3%) were screen-detected CRCs and 4018 (26.2%) were interval CRCs identified through December 31, 2019 (eFigure 1 in Supplement 1). Missing data for identified CRC were approximately 1% in national cancer and death registries. Of the patients with interval CRC (n = 4018), 2782 (69.2%; mean [SD] age at diagnosis, 62.0 [5.7] years; 1336 females [48.0%] and 1446 males [52.0%]) had post-FIT interval CRC and 1236 (30.8%; mean [SD] age at diagnosis, 64.2 [6.0] years; 564 females [45.6%] and 672 males [54.4%]) had postcolonoscopy interval CRC, with most postcolonoscopy interval CRC cases in the low- and middle-ADR groups. Proximal CRCs were more common in postcolonoscopy interval CRC than in post-FIT interval CRC (408 [33.0%] vs 776 [27.9%]; *P* = .001) (Table 1). The stage distributions of interval CRC are shown in eFigure 2 in Supplement 1.

**Table 1.** Incidence, Mortality Rate, and Demographics of Individuals With Interval CRC in the Taiwan CRC Screening Program

Variable	Post-FIT interval CRC	Postcolonoscopy interval CRC	Postcolonoscopy interval CRC by hospital-level ADR		
			High ADR^a^	Middle ADR^a^	Low ADR^a^
Population, No.	2 810 352	172 536	50 010	83 742	38 784
Person-year	29 462 192.4	1 652 673.9	484 425.6	759 595.6	408 652.7
CRC cases, No.	2782	1236	146	533	557
Incidence rate per 1000 person-years (95% CI)	0.09 (0.09-0.10)	0.75 (0.71-0.79)	0.30 (0.25-0.35)	0.70 (0.64-0.76)	1.36 (1.24-1.47)
Age at diagnosis, No. (%)					
50-59 y	1057 (38.0)	349 (28.2)	40 (27.4)	140 (26.3)	169 (30.3)
≥60 y	1725 (62.0)	887 (71.8)	106 (72.6)	393 (73.7)	388 (69.7)
Sex, No. (%)					
Female	1336 (48.0)	564 (45.6)	59 (40.4)	254 (47.7)	251 (45.0)
Male	1446 (52.0)	672 (54.4)	87 (59.6)	279 (52.3)	306 (55.0)
Location of cancer, No. (%)					
Distal	2006 (72.1)	828 (67.0)	82 (56.2)	362 (67.9)	384 (68.9)
Proximal	776 (27.9)	408 (33.0)	64 (43.8)	171 (32.1)	173 (31.1)
CRC-specific death, No. (%)	724 (26.0)	211 (17.1)	31 (21.2)	100 (18.7)	80 (14.3)
CRC-specific mortality per 1000 person-years (95% CI)	0.02 (0.02-0.03)	0.12 (0.11-0.14)	0.06 (0.04-0.09)	0.13 (0.09-0.16)	0.20 (0.16-0.23)

Abbreviations: ADR, adenoma detection rate; CRC, colorectal cancer; FIT, fecal immunochemical test.

^a^ Low ADR is defined as less than 40%, middle ADR as 40% to less than 65%, and high ADR as 65% or higher.

The post-FIT interval CRC incidence was 0.09 (95% CI, 0.09-0.10) per 1000 person-years among those with a negative FIT result, while postcolonoscopy interval CRC incidence was 0.75 (95% CI, 0.71-0.79) per 1000 person-years among patients who underwent a colonoscopy after a positive FIT result (Table 1). In multivariable analyses, the risk of postcolonoscopy interval CRC incidence among those who underwent a colonoscopy after a positive FIT result was higher than the post-FIT interval CRC incidence among those with a negative FIT result (AHR, 7.06; 95% CI, 6.35-7.57) (eTable 1 in Supplement 1).

Post-FIT interval CRC-specific mortality was 0.02 (95% CI, 0.02-0.03) per 1000 person-years, while postcolonoscopy interval CRC-specific mortality was 0.12 (95% CI, 0.11-0.14) per 1000 person-years (Table 1). In multivariable analyses, the latter was significantly higher than the former (AHR, 5.04; 95% CI, 4.33-5.85) (eTable 1 in Supplement 1).

### Incidence and CRC-Specific Mortality of Postcolonoscopy Interval CRC by Hospital-Level ADR 

The number of hospitals performing colonoscopies rose from 187 in 2004 to 363 in 2019 (eTable 2 in Supplement 1). Furthermore, the quality of colonoscopy in the program improved, with the mean (SD) ADR increasing from 24.6% (13.5%) in 2004 to 51.3% (10.0%) in 2019 and the postcolonoscopy interval CRC incidence decreasing from 3.46 to 1.04 per 1000 person-years between 2004 and 2018 (eFigure 3 in Supplement 1). Among the 172 536 individuals who underwent a colonoscopy after a positive FIT result, 38 784 (22.5%) underwent a colonoscopy in low-ADR settings, 83 742 (48.5%) in middle-ADR settings, and 50 010 (29.0%) in high-ADR settings. Although 122 526 of 172 536 patients (71.0%) underwent the procedure in low- or middle-ADR settings, these groups accounted for 1090 of 1236 subsequent postcolonoscopy interval CRCs (88.2%) and 180 of 211 related deaths (85.3%). In contrast, the high-ADR group accounted for 146 postcolonoscopy interval CRCs (11.8%) and 31 related deaths (14.7%) (Table 1).

Postcolonoscopy interval CRC cases were 557 (3.6%) in the low-ADR group, 533 (3.5%) in the middle-ADR group, and 146 (1.0%) in the high-ADR group (eFigure 1 in Supplement 1). Postcolonoscopy interval CRC incidence was inversely associated with hospital ADR level, with the highest incidence in the low-ADR group (1.36 [95% CI, 1.24-1.47] per 1000 person-years), followed by the middle-ADR group (0.70 [95% CI, 0.64-0.76] per 1000 person-years) and high-ADR group (0.30 [95% CI, 0.25-0.35] per 1000 person-years) (Table 1). The proximal cases were more frequent in the high-ADR group (64 [43.8%]) than in the middle-ADR (171 [32.1%]) and low-ADR groups (173 [31.1%]; *P* = .01) (Table 1).

**Figure 2.**
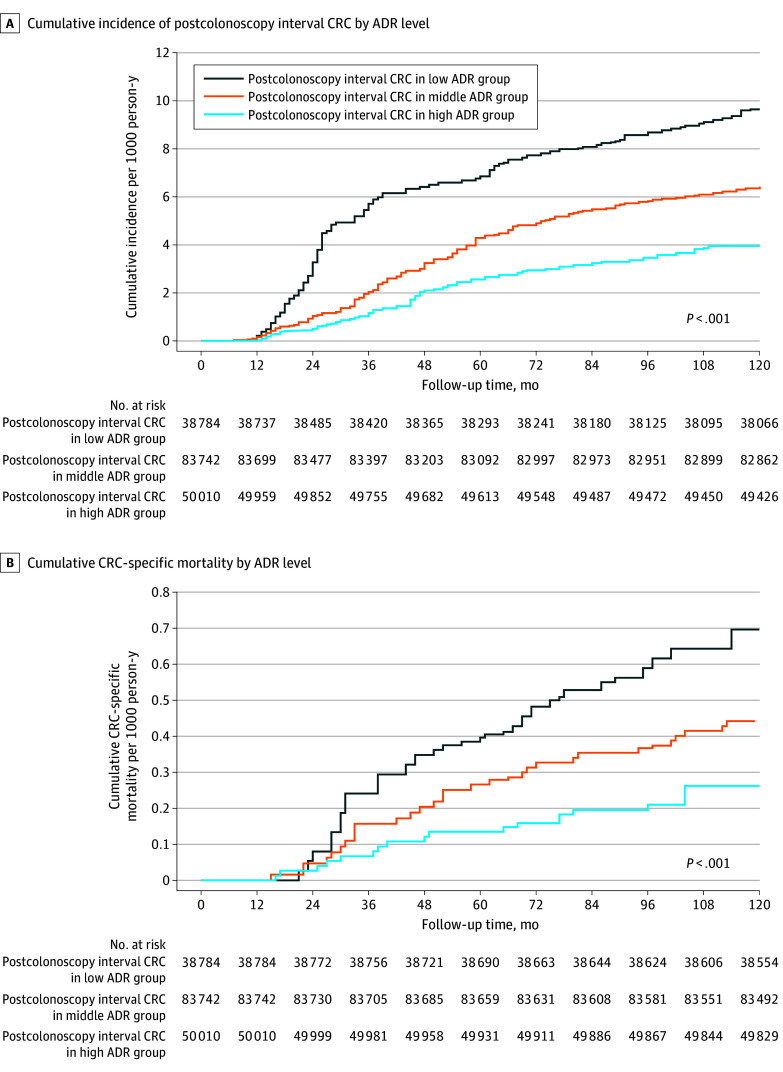
Cumulative Incidence of Postcolonoscopy Interval Colorectal Cancer (CRC) and CRC-Specific Mortality in Hospitals by Level of Adenoma Detection Rate Low adenoma detection rate (ADR) is defined as less than 40%, middle ADR as 40% to less than 65%, and high ADR as 65% or higher.

The cumulative incidence of postcolonoscopy interval CRC by different ADR groups is shown in Figure 2A. In multivariate analyses, compared with the low-ADR group, the risk of postcolonoscopy interval CRC was significantly lower in the middle-ADR group (AHR, 0.57; 95% CI, 0.43-0.72) and high-ADR group (AHR, 0.26; 95% CI, 0.20-0.36) (eTable 1 in Supplement 1). The high-ADR group also showed a significantly lower risk than the middle-ADR group (AHR, 0.46; 95% CI, 0.33-0.61).

The CRC-specific mortality for postcolonoscopy interval CRC was 0.20 (95% CI, 0.16-0.23) per 1000 person-years in the low-ADR group, 0.13 (95% CI, 0.09-0.16) per 1000 person-years in the middle-ADR group, and 0.06 (95% CI, 0.04-0.09) per 1000 person-years in the high-ADR group (Table 1). The cumulative CRC-specific mortality by ADR groups is shown in Figure 2B. Compared with the low-ADR group, the risk of CRC-specific mortality was significantly lower in the middle-ADR (AHR, 0.65; 95% CI, 0.47-0.90) and high-ADR (AHR, 0.28; 95% CI, 0.19-0.41) groups (eTable 1 in Supplement 1). Compared with the middle-ADR group, the high-ADR group also showed significantly lower risk (AHR, 0.43; 95% CI, 0.28-0.67). The results of the sensitivity analysis adjusted for competing causes of death are shown in eTable 3 in Supplement 1.

### Survival Analysis of Different Categories of Interval CRC

**Figure 3.**
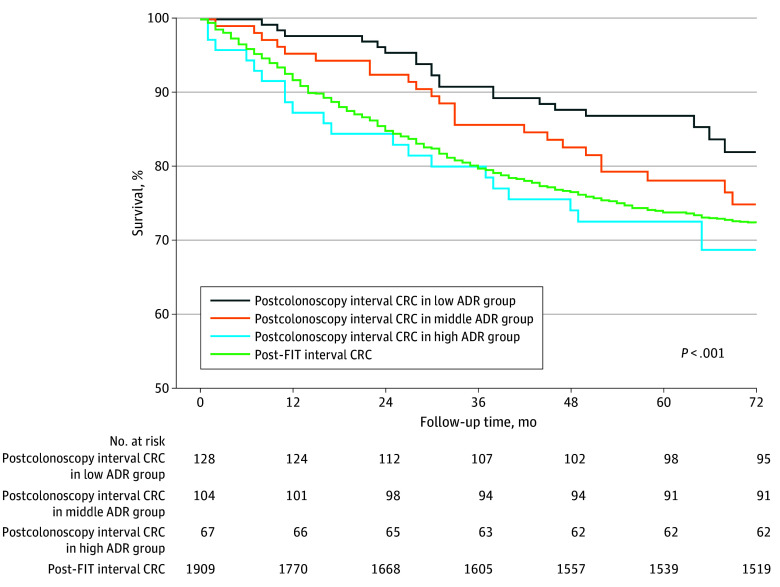
Long-Term Risk of Colorectal Cancer (CRC) Death Associated With Post–Fecal Immunochemical Test (FIT) and Postcolonoscopy Interval CRC With Various Hospital-Level Adenoma Detection Rates Low adenoma detection rate (ADR) is defined as less than 40%, middle ADR as 40% to less than 65%, and high ADR as 65% or higher.

A total of 2394 interval CRC cases were identified through December 31, 2012. Of those cases, 2078 (86.8%) were post-FIT interval CRC and 316 (13.2%) were postcolonoscopy interval CRC. During a mean (SD) follow-up of 5.72 (3.15) years after interval CRC diagnosis, a total of 672 CRC deaths (28.1%) were observed (eTable 4 in Supplement 1). The survival rates of post-FIT interval CRC and postcolonoscopy interval CRC across different ADR levels differed significantly (eg, 5-year survival rate for postcolonoscopy interval CRC in the high-ADR group vs the low-ADR group: 68.7% vs 82.0%; *P* < .001) (Figure 3). After stratification, the 5-year survival rate after postcolonoscopy interval CRC diagnosis was the worst in the high-ADR group (68.7%). In multivariable analyses, the AHR for CRC-specific death was 1.32 (95% CI, 1.03-1.69) for post-FIT interval CRC compared with postcolonoscopy interval CRC (Table 2). However, no significant difference was found between the middle-ADR and high-ADR groups. The risk of CRC-specific death was significantly higher for postcolonoscopy interval CRC in the high-ADR group than in the low-ADR group (AHR, 1.89; 95% CI, 1.04-3.43). Sensitivity analyses for the follow-up durations and stratified analyses by index colonoscopy findings are presented in eTables 5 and 6, respectively, in Supplement 1.

**Table 2.** Risk of Death After the Diagnosis of Interval CRC

Comparison of different interval CRC categories^a^^,^^b^^,^^c^	HR (95% CI)	
	Univariate, HR	Multivariable, AHR^d^
Post-FIT interval CRC vs postcolonoscopy interval CRC	1.35 (1.05-1.73)	1.32 (1.03-1.69)
Post-FIT interval CRC vs postcolonoscopy interval CRC in the low-ADR group	1.64 (1.09-2.47)	1.92 (1.28-2.89)
Post-FIT interval CRC vs postcolonoscopy interval CRC in the middle-ADR group	1.23(0.81-1.76)	1.36 (0.91-2.03)
Post-FIT interval CRC vs postcolonoscopy interval CRC in the high-ADR group	0.96 (0.61-1.50)	1.02 (0.65-1.59)
Postcolonoscopy interval CRC in the middle-ADR vs low-ADR group	1.26 (0.71-2.23)	1.42 (0.81-2.48)
Postcolonoscopy interval CRC in the high-ADR vs low-ADR group	1.64 (0.90-2.97)	1.89 (1.04-3.43)
Postcolonoscopy interval CRC in the high-ADR vs middle-ADR group	1.26 (0.69-2.27)	1.34 (0.74-2.41)

Abbreviations: ADR, adenoma detection rate; AHR, adjusted hazard ratio; CRC, colorectal cancer; FIT, fecal immunochemical test; HR, hazard ratio.

^a^ Overall *P* value for interval CRC category (3 *df*): *P* = .01.

^b^ Diagnosis interval: 0 to 3 years, 3 to 5 years, and more than 5 years from FIT or colonoscopy to interval CRC diagnosis.

^c^ Low ADR is defined as less than 40%, middle ADR as 40% to less than 65%, and high ADR as 65% or higher.

^d^ Adjusted for age at CRC diagnosis, sex, diagnosis interval, anatomical location, and cancer stage.

## Discussion

We investigated the burden and outcomes of interval CRC in the FIT-based Taiwan CRC Screening Program. Post-FIT interval CRC constituted the majority of interval CRCs, even though the risk among those with a negative FIT result was much lower than the risk of postcolonoscopy interval CRC among those who underwent a colonoscopy after a positive FIT result. This finding is not paradoxical; far more people underwent a FIT than underwent a colonoscopy after a positive FIT result, and a lower risk among a larger number of people was associated with the majority of interval CRC.

We believe our most notable finding is the worse postcolonoscopy interval CRC survival in the high-ADR group compared with the low-ADR and middle-ADR groups, with survival similar to that of post-FIT interval CRC. This finding suggests a complex interplay between instrumental factors, such as ADR, and the intrinsic biological behavior of cancers, affecting the pattern of interval CRCs and their long-term outcomes. The poor survival observed for both postcolonoscopy interval CRC (which occurs despite high ADR) and post-FIT interval CRC suggests that these cancers may be biologically more aggressive.

Post-FIT interval CRC accounted for the majority (69.2%) of all interval CRC in our study. Although the framework of the FIT-based screening policy is difficult to change, the sensitivity of FIT for CRC and advanced adenoma could be improved by using a 2-sample FIT, lowering the cutoff value, or shortening the screening interval to 1 year.^27,28^

In the current study, ADR level was inversely associated with postcolonoscopy interval CRC incidence and CRC-specific mortality, consistent with previous cohort studies.^13,14^ However, despite lower postcolonoscopy interval CRC incidence and CRC-specific mortality in the high-ADR group, our analysis shows that an incidence of 0.30 per 1000 person-years still accounted for 11.8% of postcolonoscopy interval CRC cases and 14.7% of their related deaths. This finding may reflect the limitations of relying solely on ADR to assess colonoscopy quality and the potential ceiling of its protective benefit. Studies in different screening programs have found no significant variation in postcolonoscopy interval CRC risk across the higher ADR tiers.^10,13^ In the Dutch program, an ADR of 70% corresponded to approximately 2 postcolonoscopy interval CRC cases per 1000 colonoscopies over 5 years.^14^ These findings suggest that high ADR is crucial but may not be sufficient to ensure the highest effectiveness, indicating a potential performance plateau.

Postcolonoscopy interval CRC in the high-ADR group were more frequently located in the proximal colon and had worse survival (Table 1). A potential explanation is that subtle but clinically relevant lesions, such as flat or laterally spreading tumors and SSLs, might have been missed or inadequately resected even in high-ADR hospitals. Laterally spreading tumors or SSLs, both frequently located proximally, are more likely to be missed and lead to postcolonoscopy interval CRC.^29,30,31^ Postcolonoscopy interval CRC compared with screening-detected CRC more frequently exhibits *BRAF* sequence variation, develops proximally, and has worse survival rates.^32,33,34^ Missed SSLs likely contribute to postcolonoscopy interval CRC due to rapid CRC progression after dysplasia develops.^35^ Postcolonoscopy interval CRC may result from missed or incompletely resected polypoid lesions, harder-to-detect nonpolypoid lesions, or new lesions after colonoscopy, with varying impact across ADR levels. If detection and resection could be perfected, new lesions after colonoscopy would define the ceiling maximum for colonoscopy.

Postcolonoscopy interval CRC in the low-ADR and middle-ADR groups accounted for 88.2% of cases and 85.3% of related deaths, despite 71.0% of patients undergoing colonoscopy. Suboptimal colonoscopy quality contributes to these outcomes and may be modifiable. Image-enhanced endoscopy, distal attachment, or artificial intelligence–assisted colonoscopy may improve ADR, but their effectiveness in reducing postcolonoscopy interval CRC incidence remains unclear.^36^ Nevertheless, colonoscopy quality in the Taiwan CRC Screening Program has improved, with ADR rising from 24.6% in 2004 to 51.3% in 2019 and postcolonoscopy interval CRC incidence dropping from 3.46 to 1.04 per 1000 person-years (2004-2018) (eFigure 3 in Supplement 1). These improvements were associated with quality initiatives, standardized reporting, training, and greater public awareness, as well as compliance with precolonoscopy instructions and follow-up recommendations.

### Strengths and Limitations

This study has several strengths. First, it leverages data from the Taiwan CRC Screening Program with 2 982 888 participants, providing a considerable number of post-FIT interval CRC and postcolonoscopy interval CRC cases for precise risk estimation. Second, our screening program is offered to persons with average risk aged 50 to 74 years, similar to other programs worldwide, which enhances the generalizability. Third, all interval CRC cases were identified and verified by linking to the national cancer and death registries, covering almost the entire population of Taiwan.^17^ Fourth, the extended follow-up duration enabled robust estimation of interval CRC risk after FIT and colonoscopy. In addition, we conducted sensitivity analyses adjusting for competing causes of death. The higher mortality risk after a colonoscopy than after a negative FIT result remained, while higher ADR showed a protective benefit. Furthermore, immortal time and lead time biases may affect survival measurement in postcolonoscopy interval CRC, but adjustments confirmed consistent results^37^ (eTable 7 in Supplement 1). Further sensitivity analyses of follow-up duration and colonoscopy findings showed no association with CRC death in post-FIT and postcolonoscopy interval CRC across ADR levels, reinforcing our findings.

This study has limitations. First, ADR was assessed at the hospital level rather than at the individual endoscopist level, which may have introduced bias due to variation among endoscopists within the same hospital. While hospital-level ADR is practical, it may overlook individual differences, especially in larger institutions with multiple endoscopists. Because endoscopist-level data became available only after 2015, we could not incorporate this variation into the current analysis. Second, our analysis revealed that polyp characteristics varied across hospitals by ADR categories, suggesting that case-mix differences are likely one of the explanations for the observed differences in CRC death (eTable 8 in Supplement 1). Future exploration of these case-mix factors might be possible using data from the standardized colonoscopy report format implemented in Taiwan in 2015. Third, although most cases of postcolonoscopy interval CRC occur within 2 to 3 years after colonoscopy, the follow-up period may not be adequate to detect all postcolonoscopy interval CRC cases and related deaths, particularly for persons with a negative colonoscopy result later in the study period.^38,39^ Fourth, follow-up started at the FIT date for post-FIT interval CRC and at the colonoscopy date for postcolonoscopy interval CRC. This difference could introduce bias but was likely minimal, as over 95% of individuals with a positive FIT result in the program underwent a colonoscopy within 6 months and 78% did so within 3 months of a positive FIT result.^20^

## Conclusions

In this cohort study of the FIT-based Taiwan CRC Screening Program, post-FIT interval CRC had a greater absolute incidence and mortality burden than postcolonoscopy interval CRC despite higher rates in the at-risk subpopulations for postcolonoscopy interval CRC due to the larger at-risk population for post-FIT interval CRC. The worse survival of postcolonoscopy interval CRC in high-ADR settings suggests a ceiling effect for current colonoscopic techniques and quality assurance. It also highlights the possible interplay between instrumental and biological factors that might require innovative preventive and therapeutic interventions.

## Notes

Supplement 1. **eMethods.** Hospital-Level Adenoma Detection Rate (ADR) Calculation **eFigure 1.** Attributable Proportion of Different CRCs in the Taiwan CRC Screening Program **eFigure 2.** Stage Distribution of Different Categories of CRCi in the Taiwan CRC Screening Program **eTable 1.** Comparison of the Risk of CRCi and CRC-Specific Mortality After Negative FIT and After Follow-up Colonoscopy for Positive FIT **eTable 2.** The Number of Hospitals Among Different ADR Hospitals From 2004 to 2019 **eFigure 3.** The Temporal Trends in ADR Improvement and the Incidence of Post-Colonoscopy-CRCi Within the Taiwan CRC Screening Program **eTable 3.** Comparison of the Risk of CRC-Specific Mortality After Negative FIT and After Follow-up Colonoscopy for Positive FIT Without and With Adjustment for Competing Causes of Death **eTable 4.** Numbers and 5-year Survival Rates of Post-FIT-CRCi and Post-Colonoscopy-CRCi **eTable 5.** Sensitivity Analyses With Standardized Follow-up Durations **eTable 6.** Risk of CRC Death After the Diagnosis of CRCi **eTable 7.** Adjusting for Lead Time and Immortal Time Biases for the Risk of CRC Death After the Diagnosis of CRCi **eTable 8.** Polyp Characteristics of Index Colonoscopy Across Hospitals With Different ADR Categories

Supplement 2.
Data Sharing Statement
