# Supplementary material for: Interval Colorectal Cancers in a Fecal Immunochemical Test–Based Screening Program
Source: JAMA Netw Open. 2025 Jul 28;8(7):e2523441. doi: 10.1001/jamanetworkopen.2025.23441 (PMC12305388; doi:10.1001/jamanetworkopen.2025.23441)
Supplement: Supplement 1. — eMethods. Hospital-Level Adenoma Detection Rate (ADR) Calculation eFigure 1. Attributable Proportion of Different CRCs in the Taiwan CRC Screening Program eFigure 2. Stage Distribution of Different Categories of CRCi in the Taiwan CRC Screening Program eTable 1. Comparison of the Risk of CRCi and CRC-Specific Mortality After Negative FIT and After Follow-up Colonoscopy for Positive FIT eTable 2. The Number of Hospitals Among Different ADR Hospitals From 2004 to 2019 eFigure 3. The Temporal Trends in ADR Improvement and the Incidence of Post-Colonoscopy-CRCi Within the Taiwan CRC Screening Program eTable 3. Comparison of the Risk of CRC-Specific Mortality After Negative FIT and After Follow-up Colonoscopy for Positive FIT Without and With Adjustment for Competing Causes of Death eTable 4. Numbers and 5-year Survival Rates of Post-FIT-CRCi and Post-Colonoscopy-CRCi eTable 5. Sensitivity Analyses With Standardized Follow-up Durations eTable 6. Risk of CRC Death After the Diagnosis of CRCi eTable 7. Adjusting for Lead Time and Immortal Time Biases for the Risk of CRC Death After the Diagnosis of CRCi eTable 8. Polyp Characteristics of Index Colonoscopy Across Hospitals With Different ADR Categories [file jamanetwopen-e2523441-s001.pdf]

Supplemental Online Content

Hsu W-F, Ladabaum U, Su C-W, et al. Interval colorectal cancers in a fecal immunochemical test–based screening program. *JAMA Netw Open*. 2025;8(7):e2523441. doi:10.1001/jamanetworkopen.2025.23441

eMethods. Hospital-Level Adenoma Detection Rate (ADR) Calculation
eFigure 1. Attributable Proportion of Different CRCs in the Taiwan CRC Screening Program
eFigure 2. Stage Distribution of Different Categories of CRCi in the Taiwan CRC Screening Program
eTable 1. Comparison of the Risk of CRCi and CRC-Specific Mortality After Negative FIT and After Follow-up Colonoscopy for Positive FIT
eTable 2. The Number of Hospitals Among Different ADR Hospitals From 2004 to 2019
eFigure 3. The Temporal Trends in ADR Improvement and the Incidence of Post-Colonoscopy-CRCi Within the Taiwan CRC Screening Program
eTable 3. Comparison of the Risk of CRC-Specific Mortality After Negative FIT and After Follow-up Colonoscopy for Positive FIT Without and With Adjustment for Competing Causes of Death
eTable 4. Numbers and 5-year Survival Rates of Post-FIT-CRCi and Post-Colonoscopy-CRCi
eTable 5. Sensitivity Analyses With Standardized Follow-up Durations
eTable 6. Risk of CRC Death After the Diagnosis of CRCi
eTable 7. Adjusting for Lead Time and Immortal Time Biases for the Risk of CRC Death After the Diagnosis of CRCi
eTable 8. Polyp Characteristics of Index Colonoscopy Across Hospitals With Different ADR Categories

This supplemental material has been provided by the authors to give readers additional information about their work.

© 2025 Hsu W-F et al. *JAMA Network Open*

eMethods. Hospital-level adenoma detection rate (ADR) calculation

We calculated ADR based on colonoscopy procedures performed after a positive FIT result within the program. Since we could not access individual endoscopist-level ADR data during the study period, we utilized hospital-level ADR instead. Due to the dynamic nature of hospital-level ADR, which can be influenced by the composition of endoscopists within each hospital, we calculated the ADR for each hospital every three years to accommodate this variability, ensuring each hospital performed at least 100 colonoscopies for accurate classification.

© 2025 Hsu W-F et al. *JAMA Network Open*

eFigure 1. Attributable proportion of different CRCs in the Taiwan CRC Screening Program

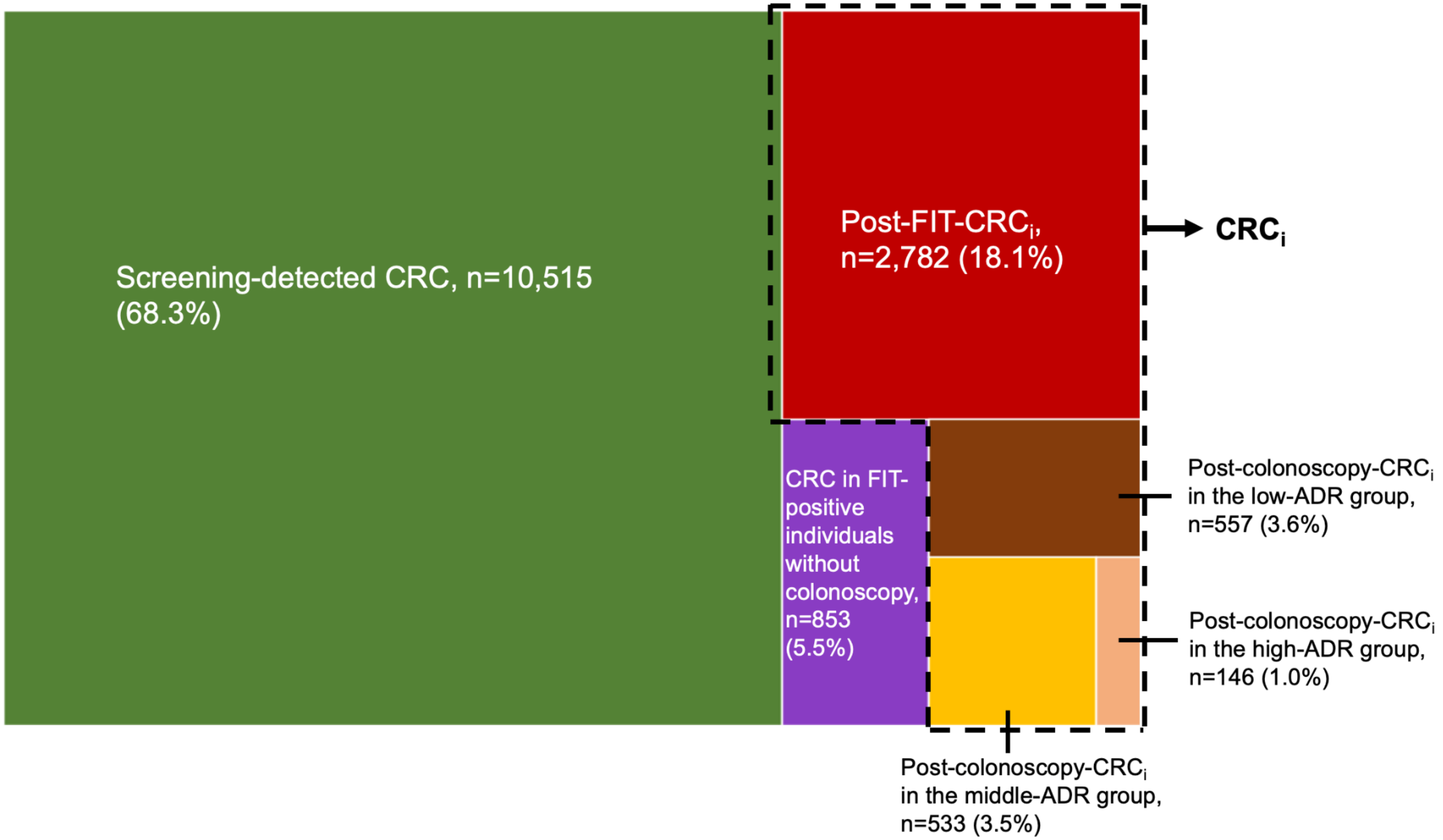

ADR, adenoma detection rate; CRC, colorectal cancer; CRC_i, interval colorectal cancer; FIT, fecal immunochemical test; Post-colonoscopy-CRC_i, interval type post-colonoscopy colorectal cancer; Post-FIT-CRC_i, interval type post-FIT colorectal cancer.
Low ADR: ADR<40%; middle ADR: 40%≤ ADR<65%; high ADR: 65%≤ADR

© 2025 Hsu W-F et al. *JAMA Network Open*

eFigure 2. Stage distribution of different categories of CRC_i in the Taiwan CRC Screening Program

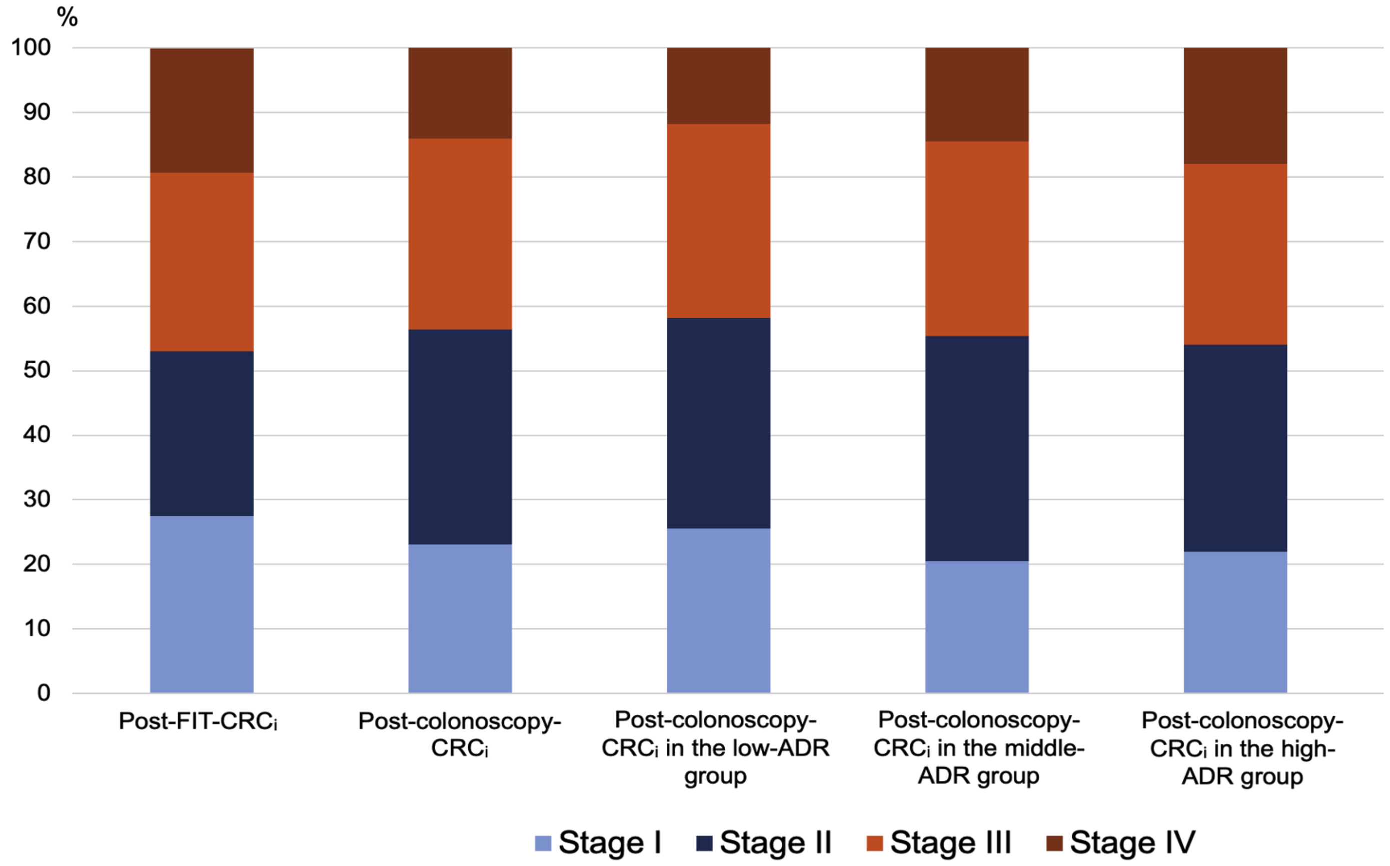

ADR, adenoma detection rate; CRC, colorectal cancer; Post-colonoscopy-CRC_i, interval type post-colonoscopy colorectal cancer.
Low ADR: ADR<40%; middle ADR: 40%≤ ADR<65%; high ADR: 65%≤ADR

© 2025 Hsu W-F et al. *JAMA Network Open*

eTable 1. Comparison of the risk of CRC_i and CRC-specific mortality after negative FIT and after follow-up colonoscopy for positive FIT

	Risk of CRC_i*	Risk of CRC-specific mortality**
	aHR(95%CI)	aHR(95%CI)
**CRC_i**		
After colonoscopy (n= 172,536) vs. after negative FIT (n=2,810,352)	7.06 (6.35-7.57)	5.04 (4.33-5.85)
**Post-colonoscopy-CRC_is**		
Middle ADR (n=83,742) vs. low ADR (n=38,784)	0.57 (0.43-0.72)	0.65 (0.47-0.90)
High ADR (n=50,010) vs. low ADR (n=38,784)	0.26 (0.20-0.36)	0.28 (0.19-0.41)
High ADR (n=50,010) vs. middle ADR (n= 83,742)	0.46 (0.33-0.61)	0.43 (0.28-0.67)

*Multivariable analysis: adjusted for age at screening and sex.
** Multivariable analysis: adjusted for age at screening, sex, and
ADR: adenoma detection rate; aHR, adjusted hazard ratio; CI: confidence interval; CRC: colorectal cancer; CRC_i: interval colorectal cancer; Post-colonoscopy-CRC_i, interval type post-colonoscopy colorectal cancer; Post-FIT-CRC_i: interval type post-FIT colorectal cancer.
Low ADR: ADR<40%; middle ADR: 40%≤ ADR<65%; high ADR: 65%≤ADR

eTable 2. The number of hospitals among different ADR hospitals from 2004 to 2019

Year range	Total hospital, N	High ADR hospitals, n	Middle ADR hospitals, n	Low ADR hospitals, n	Total Colonoscopies, n	Colonoscopies in high ADR hospital, n (%)	Colonoscopies in middle ADR hospital, n (%)	Colonoscopies in low ADR hospital, n (%)
**2004-2006**	187	2	62	123	16,306	967 (5.9%)	2,010 (12.3%)	13,329 (81.7%)
**2007-2009**	210	9	76	125	25,411	4,312 (17.0%)	9,768 (38.4%)	11,311 (44.5%)
**2010-2012**	282	21	157	104	136,759	37,692 (27.6%)	72,834 (53.3%)	26,233 (19.2%)
**2013-2015**	352	33	197	122	165,369	46,247 (28.0%)	87,864 (53.1%)	21,258 (12.9%)
**2016-2019**	363	52	220	91	212,419	68,328 (41.3%)	123,062 (74.4%)	21,029 (12.7%)

ADR, adenoma detection rate.
Note: Since 2019 only has one year of data, it is combined with the data from 2016-2018 to calculate the ADR.

© 2025 Hsu W-F et al. *JAMA Network Open*

eFigure 3. The temporal trends in ADR improvement and the incidence of post-colonoscopy-CRC_i within the Taiwan CRC Screening Program

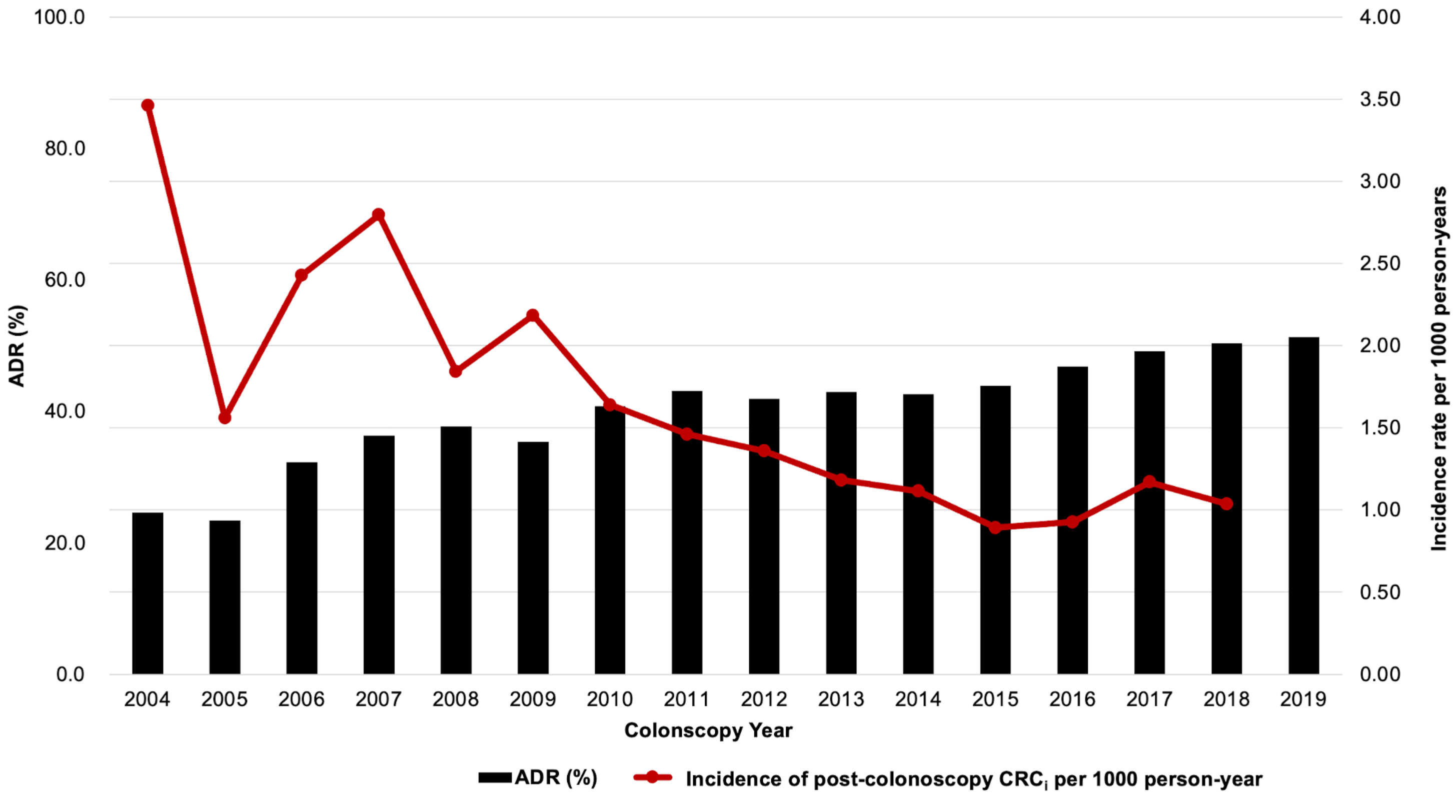

ADR: adenoma detection rate; CRC_i: interval colorectal cancer; Post-colonoscopy-CRC_i, interval type post-colonoscopy colorectal cancer.

© 2025 Hsu W-F et al. *JAMA Network Open*

eTable 3. Comparison of the risk of CRC-specific mortality after negative FIT and after follow-up colonoscopy for positive FIT without and with adjustment for competing causes of death

	Cause-specific hazards	Subdistribution hazards
	aHR(95%CI)	aHR(95%CI)
**CRC_i**		
After colonoscopy (n= 172,536) vs. after negative FIT (n=2,810,352)	5.04 (4.33-5.85)	7.65 (7.36-7.95)
**Post-colonoscopy-CRC_is**		
Middle ADR (n=83,742) vs. low ADR (n=38,784)	0.65 (0.47-0.90)	0.58 (0.53-0.63)
High ADR (n=50,010) vs. low ADR (n=38,784)	0.28 (0.19-0.41)	0.37 (0.31-0.43)
High ADR (n=50,010) vs. middle ADR (n= 83,742)	0.43 (0.28-0.67)	0.66 (0.60-0.74)

The overall p-value for post-colonoscopy-CRC_i subgroup comparisons (low, middle, and high ADR) was < 0.001.
ADR: adenoma detection rate; aHR, adjusted hazard ratio; CI: confidence interval; CRC: colorectal cancer; CRC_i: interval colorectal cancer; Post-colonoscopy-CRC_i, interval type post-colonoscopy colorectal cancer; Post-FIT-CRC_i: interval type post-FIT colorectal cancer.
Low ADR: ADR<40%; middle ADR: 40%≤ ADR<65%; high ADR: 65%≤ADR

© 2025 Hsu W-F et al. *JAMA Network Open*

eTable 4. Numbers and 5-year survival rates of post-FIT-CRC_i and post-colonoscopy-CRC_i

Detection mode	Post-FIT-CRC_i, n (%)	Post-colonoscopy-CRC_i	Post-colonoscopy-CRC_i, n (%)		
			High-ADR group	Middle-ADR group	Low-ADR group
**Total cases**	2078	316	72	109	135
**Mean follow-up time, years**	5.9	5.7	5.9	5.7	5.5
**CRC deaths, n (%)**	602 (29.0)	70(22.2)	21 (29.2)	25 (22.9)	24 (17.8)
**Death rate, per 1,000 person-years**	49.1	38.9	49.4	40.2	32.3

ADR: adenoma detection rate; CRC: colorectal cancer; Post-colonoscopy-CRC_i, interval type post-colonoscopy colorectal cancer; Post-FIT-CRC_i: interval type post-FIT colorectal cancer.
Low ADR: ADR<40%; middle ADR: 40%≤ ADR<65%; high ADR: 65%≤ADR

© 2025 Hsu W-F et al. *JAMA Network Open*

eTable 5. Sensitivity analyses with standardized follow-up durations

Comparison of different CRC_i Categories	Multivariable	
	Conventional method	Standardized follow-up at 5 years
	aHR (95% CI)	aHR (95% CI)
Post-FIT-CRC_i vs. post-colonoscopy-CRC_i	1.32 (1.03-1.69)	1.31 (1.02-1.68)
Post-FIT-CRC_i (vs. post-colonoscopy-CRC_i in the low-ADR group)	1.92 (1.28-2.89)	1.87 (1.24-2.70)
Post-FIT-CRC_i (vs. post-colonoscopy-CRC_i in the middle-ADR group)	1.36 (0.91-2.03)	1.22 (0.82-1.83)
Post-FIT-CRC_i (vs. post-colonoscopy-CRC_i in the high-ADR group)	1.02 (0.65-1.59)	1.03 (0.66-1.62)
Post-colonoscopy-CRC_i in the middle-ADR group (vs. post-colonoscopy-CRC_i in the low-ADR group)	1.42 (0.81-2.48)	1.38 (0.76-2.34)
Post-colonoscopy-CRC_i in the high-ADR group (vs. post-colonoscopy-CRC_i in the low-ADR group)	1.89 (1.04-3.43)	1.85 (1.00-3.38)
Post-colonoscopy-CRC_i in the high-ADR group (vs. post-colonoscopy-CRC_i in the middle-ADR group)	1.34 (0.74-2.41)	1.29 (0.66-2.38)

ADR: adenoma detection rate; aHR, adjusted hazard ratio; CI: confidence interval; CRC: colorectal cancer; CRC_i: interval colorectal cancer; Post-colonoscopy-CRC_i, interval type post-colonoscopy colorectal cancer; Post-FIT-CRC_i: interval type post-FIT colorectal cancer.
Low ADR: ADR<40%; middle ADR: 40%≤ ADR<65%; high ADR: 65%≤ADR

© 2025 Hsu W-F et al. *JAMA Network Open*

eTable 6. Risk of CRC death after the diagnosis of CRC_i

Comparison of different CRC_i Categories	Multivariable			
	All colonoscopy	No neoplasm	Without advanced adenomas	With advanced adenomas
	aHR (95% CI)	aHR (95% CI)	aHR (95% CI)	aHR (95% CI)
Post-colonoscopy-CRC_i in the middle-ADR group (vs. post-colonoscopy-CRC_i in the low-ADR group)	1.42 (0.81-2.48)	1.75 (0.87-3.53)	1.76 (0.67-4.24)	2.16 (0.52-9.38)
Post-colonoscopy-CRC_i in the high-ADR group (vs. post-colonoscopy-CRC_i in the low-ADR group)	1.89 (1.04-3.43)	2.21 (1.04-4.67)	2.34(1.07-6.85)	2.84 (0.75-14.73)
Post-colonoscopy-CRC_i in the high-ADR group (vs. post-colonoscopy-CRC_i in the middle-ADR group)	1.34 (0.74-2.41)	1.26 (0.63-2.53)	1.33 (0.67-7.48)	1.37 (0.52-9.98)

ADR: adenoma detection rate; aHR, adjusted hazard ratio; CI: confidence interval; CRC: colorectal cancer; CRC_i: interval colorectal cancer; Post-colonoscopy-CRC_i, interval type post-colonoscopy colorectal cancer; Post-FIT-CRC_i: interval type post-FIT colorectal cancer.
Low ADR: ADR<40%; middle ADR: 40%≤ ADR<65%; high ADR: 65%≤ADR

© 2025 Hsu W-F et al. *JAMA Network Open*

eTable 7. Adjusting for lead time and immortal time biases for the risk of CRC death after the diagnosis of CRC_i

Comparison of different CRC_i categories	Multivariable	
	Analysis 1* aHR (95% CI)	Analysis 2** aHR (95% CI)
Post-colonoscopy-CRC_i in the middle-ADR group (vs. post-colonoscopy-CRC_i in the low-ADR group)	1.42 (0.81-2.48)	1.56 (0.97-3.12)
Post-colonoscopy-CRC_i in the high-ADR group (vs. post-colonoscopy-CRC_i in the low-ADR group)	1.89 (1.04-3.43)	2.26 (1.33-3.96)
Post-colonoscopy-CRC_i in the high-ADR group (vs. post-colonoscopy-CRC_i in the middle-ADR group)	1.34 (0.74-2.41)	1.69 (1.08-2.76)

*Analysis 1: Survival measured from CRC diagnosis date (conventional method).

**Analysis 2: Survival measured from 36 months after the index colonoscopy, addressing both lead time and immortal time biases.

ADR: adenoma detection rate; CRC: colorectal cancer; CRC_i: interval colorectal cancer; Post-colonoscopy-CRC_i, interval type post-colonoscopy colorectal cancer.
Low ADR: ADR<40%; middle ADR: 40%≤ ADR<65%; high ADR: 65%≤ADR

© 2025 Hsu W-F et al. *JAMA Network Open*

eTable 8. Polyp characteristics of index colonoscopy across hospitals with different ADR categories

Hospitals with different ADR	Subjects with advanced adenoma, n (%)	Subjects with non-advanced adenoma	p-value
High ADR hospitals	13,283 (39.1%)	20,718 (60.9%)	p<0.001
Middle ADR hospitals	14,248 (36.2%)	25,110 (64.8%)	
Low ADR hospitals	3,417 (33.8%)	6,667 (66.2%)	

ADR: adenoma detection rate
Low ADR: ADR<40%; middle ADR: 40%≤ ADR<65%; high ADR: 65%≤ADR

© 2025 Hsu W-F et al. *JAMA Network Open*
