# Supplementary material for: Interval Colorectal Cancers in a Fecal Immunochemical Test–Based Screening Program
Source: JAMA Netw Open. 2025 Jul 28;8(7):e2523441. doi: 10.1001/jamanetworkopen.2025.23441 (PMC12305388; doi:10.1001/jamanetworkopen.2025.23441)
Supplement: Supplement 2. — Data Sharing Statement [file jamanetwopen-e2523441-s002.pdf]

Data Sharing Statement

Hsu. Burden and Outcomes of Interval Colorectal Cancers in a Fecal Immunochemical Test–Based Screening Program. *JAMA Netw Open*. Published July 28, 2025. doi:10.1001/jamanetworkopen.2025.23441

Data
Data available: No
